# fNIRS for Tracking Brain Development in the Context of Global Health Projects

**DOI:** 10.3390/photonics6030089

**Published:** 2019-08-02

**Authors:** Anna Blasi, Sarah Lloyd-Fox, Laura Katus, Clare E. Elwell

**Affiliations:** 1 Department of Medical Physics and Biomedical Engineering, University College London, London WC1E 6BT, UK; 2 Centre for Brain and Cognitive Development, Birkbeck, University of London, London WC1E 7HX, UK; 3 Department of Psychology, University of Cambridge, Cambridge CB2 3EB, UK; 4 Great Ormond Street Institute of Child Health, University College London, London WC1N 1EH, UK

**Keywords:** fNIRS, global health, infant brain development

## Abstract

Over the past 25 years, functional near-infrared spectroscopy (fNIRS) has emerged as a valuable tool to study brain function, and it is in younger participants where it has found, arguably, its most successful application. Thanks to its infant-friendly features, the technology has helped shape research in the neurocognitive development field by contributing to our understanding of the neural underpinnings of sensory perception and socio-cognitive skills. Furthermore, it has provided avenues of exploration for markers of compromised brain development. Advances in fNIRS instrumentation and methods have enabled the next step in the evolution of its applications including the investigation of the effects of complex and interacting socio-economic and environmental adversities on brain development. To do this, it is necessary to take fNIRS out of well-resourced research labs (the majority located in high-income countries) to study at-risk populations in resource-poor settings in low- and middle-income countries (LMICs). Here we review the use of this technology in global health studies, we discuss the implementation of fNIRS studies in LMICs with a particular emphasis on the Brain Imaging for Global Health (BRIGHT) project, and we consider its potential in this emerging field.

## 1. Introduction

Over the past 25 years functional near-infrared spectroscopy (fNIRS) has become a valuable non-invasive technology to study brain function. One of the most useful features of fNIRS for neurocognitive research is its capacity to measure brain function in naturalistic settings. fNIRS offers remarkable flexibility for protocols designed to investigate the role of cortical brain networks in processing a wide range of stimuli with different levels of complexity and with participants of different ages [1]. It is in the younger-aged cohorts where fNIRS has seen, arguably, its most successful application, as its ease of use, portability, safety and, in particular, its tolerance of motion, make it an ideal technique to study neurodevelopment [2,3].

As the number of fNIRS publications on neurodevelopment continues to grow, so does the range of topics it is being used to investigate. For example, the past year has seen the publication of fNIRS infant studies focusing on speech processing [4,5], social perception and interaction [6], and face perception [7,8], alongside studies investigating more complex processing networks such as mimicry and self-perception [9,10], touch [11–14] and live interaction [15,16].

Over the last decade, researchers have also begun to use fNIRS to focus their investigations on early neural indicators of atypical development [17]. There is an increasing number of fNIRS studies collecting neuroimaging data from infants with increased familial likelihood for later diagnosis of a disorder such as autism spectrum disorder [18–20], or with other risk factors such as prematurity [21] or Down syndrome [22]. Importantly, by following the same participants longitudinally [23], researchers can begin to explore markers that help identify discrepancies between typical and atypical developmental trajectories before the onset of behavioral traits. Furthermore, this research pathway could allow the identification and implementation of protective factors that enable some infants to follow a more typical developmental trajectory despite the presence of risk factors. A myriad of factors can compromise child development, from genetic and/or environmental to complex psychosocial disadvantage. This concept of “risk” has been used interchangeably with “childhood adversity”, which Jensen et al. [24] defined as a set of “experiences or exposures that can disrupt a child’s healthy neural development and affect the achievement of early developmental milestones, language development, and cognitive capacities ( ... ) (e.g., attention, working memory, problem solving) and intelligence”. These exposures can have complex interactions and their effects on brain development are not clearly understood.

The experience accumulated by developmental scientists with fNIRS on studying brain development of typical and atypical infants, in combination with the advances in instrumentation and methods, have enabled the next step in the evolution of its applications including the investigation of the effects of childhood adversity on brain development. To do this, it is necessary to collect data from infants who are exposed to these risk factors and take fNIRS to them, out of well-resourced research labs (the majority located in high-income countries), as most of the affected populations are living in resource-poor settings in low- and middle-income countries (LMICs).

Here we review the use of fNIRS in global health studies with a particular emphasis on the Brain Imaging for Global Health (BRIGHT) project (described in more detail in Section 3). We refer to the findings from the three studies published to date to discuss the potential for this technology in this emerging field.

## 2. fNIRS to Study Effects of Early Childhood Adversity

Exposure to economic and environmental adversity has a strong impact on infant development. In 2016 McCoy and colleagues [25] estimated that almost one-third of children living in LMICs were not meeting basic milestones of cognitive and/or socio-emotional development, and that an additional 16.7% experienced delayed physical growth (stunting). The largest proportion of affected infants are living in sub-Saharan Africa.

Economic adversity, beyond restricted access to financial resources, exposes children to a complex combination of biological risks in a context of psychosocial disadvantage, forming a barrage of interactions that are difficult to disentangle [24]. One of the proposed methods to begin to understand the effect of this complex combination of factors relies on the use of biomarker data to define typical and atypical developmental pathways and measure the effectiveness of interventions [24,26]. This biomarker data can come from different modalities. Long before the emergence of neuroimaging, infant research relied mainly on observation of behavior. The use of behavioral measures in conjunction with imaging not only relates newer results with previous research, it also allows us to connect brain function with the acquisition of specific skills [27]. The majority of behavioral assessment tools have been designed and applied in U.S. or European research but work has been ongoing to adapt them for use in a much wider range of cultural contexts [28–31]. In line with this, a toolkit prepared for the Strategic Impact Evaluation Fund of the World Bank [32] offers guidelines for selection and adaptation of child development measures for use in low- and middle-income countries, and mentions fNIRS as one of the developing technologies creating new possibilities for assessing children in these settings. These new possibilities may include the combination of behavior and imaging data to disentangle the complex cause and effect pathways that link economic and environmental adversity with developmental outcome. Moreover, it is important to note that the concept of childhood adversity is not constrained to a specific set of risk factors. For example, related to the effects of under-nutrition, it is possible that infants from two different populations may have been exposed to under-nutrition of different origin, such as food with poor nutritional value or, in a different population, exposure to illness and inflammation. Therefore, to gain understanding of the effects of under-nutrition, it is essential to assess infants exposed to adversities of different origin, and therefore, it is essential to establish collaborative projects that collect data in different populations.

In 2013, one of the authors (Elwell) established Globalfnirs (www:globalfnirs.org) as a multidisciplinary platform to provide information and advice on the use of fNIRS to supplement current methods for assessing cognitive function of the developing brain in global health projects. This initiative was prompted by the successful piloting of fNIRS to study infant brain function in a field station in a rural village, Keneba, part of the Medical Research Council Unit The Gambia at the London School for Hygiene and Tropical Medicine (MRCG at LSHTM). This study delivered the first functional brain imaging of infants in Africa. Data were successfully collected from a cohort of 24 4 to 8-month old infants and the results replicated the same patterns of activation observed at similar ages in the UK with the same experimental protocol [33]. These encouraging results helped unlock the potential for fNIRS research in resource-poor communities: the technology can be packed and transported, it is easy and quick to set up, and, with basic training of research assistants, data can be readily collected and quality checks can be performed almost immediately. In a follow-up study, the team returned to the same community and collected more data, some from the same participants at 9 to 13 months and at 12 to 18 months longitudinally, plus two additional cohorts at 0 to 2 months and at 18 to 24 months. Results showed robust developmental patterns of the infant responses to social cues and further proved the validity and prospective use of fNIRS in these contexts [34]. The success of these proof-of-concept studies (see also [35,36]) demonstrated the feasibility of moving fNIRS beyond highly resourced settings to assess the impact of adversity within a global health context.

## 3. Current Studies Implementing fNIRS in Low- and Middle-Income Countries (LMICs)

Following on from these initial studies in The Gambia, funding was secured to launch the Brain Imaging for Global Health (BRIGHT) Project with the aim to establish brain function-for-age curves in infants from high- (the UK) and low-resource (The Gambia) settings in order to gain an insight into the effects that issues related to living in a low-resource context may have on infant development. This is a longitudinal study with families recruited antenatally and studied until the infant’s second birthday. Protocols are being run in parallel in the UK (with n = 62 families studied at the Rosie Hospital, Cambridge University Hospitals National Health Service Foundation Trust) and in The Gambia (with a postnatal cohort of n = 214 families studied at MRCG at LSHTM: note a further 8 families contributed antenatal data but experienced a stillbirth). The partnership between the Gambian government and the MRCG was established over 70 years ago to investigate the relationship between under-nutrition and poverty, and growth. Therefore, the interest in conducting the study there is to be able to recruit some of the affected infants into the fNIRS protocol. The project collects neuroimaging measurements, including fNIRS and electroencephalography (EEG); eye tracking; population-specific neurocognitive developmental measures (i.e., Mullen Scales of Early Learning (MSEL) and the Communicative Development Inventory (CDI)); as well as family-caregiving assessments (i.e., caregiver–infant interaction videos and questionnaires), measures of physical growth, and biological, socioeconomic, parental health and nutritional data at both sites. Although the study is still ongoing, the first results are starting to delineate distinct patterns of brain response within each population. A novel culturally appropriate paradigm tests habituation in the neural responses to repeated stimuli presentation and detection of novelty at 5 and 8 months. In the UK, a signature of habituation is reported and, with the presentation of a novel condition, a recovery response is detected. In the Gambian cohort, the habituation phase presents a similar pattern, however, at 5 and 8 months of age, the recovery phase is not yet evident [37].

**Figure 1:**
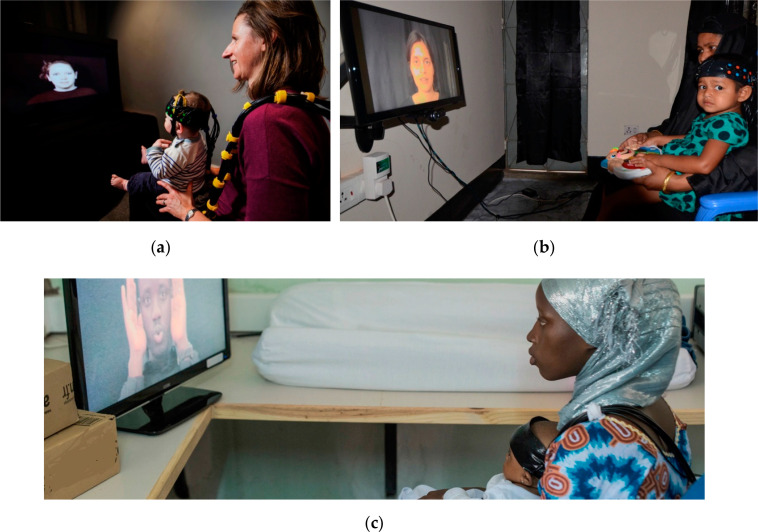
One of the paradigms shared between the BRIGHT and BEAN projects as presented in (**a**) the UK; (**b**) Bangladesh; (**c**) The Gambia.

Globalfnirs studies have also recently been reported from Bangladesh and India. Perdue et al. report results from the fNIRS component of the Bangladesh Early Adversity Neuroimaging Project [38] (BEAN, www.lcn-bean.org), another initiative that shares with the BRIGHT project the objective of studying the association between exposure to early adversities and brain development. Infants recruited into this study would have been exposed to different ranges of poverty and poverty-associated effects. The study also includes magnetic resonance imaging (MRI) and EEG as brain imaging modalities and the collection of similar infant behavioral assessments and socio-economic/nutritional/biological/ health information. It is established within an urban slum in the city of Dhaka, Bangladesh. There is an overlap in instrumentation (the NTS optical topography system, Gowerlabs Ltd. London, UK) and shared paradigms between the BEAN and BRIGHT projects. One of the common paradigms has been previously used in high-income [39,40] and LMICs [33,34] to study processing of social and non-social stimuli in the visual and auditory domains (see Figure 1 for an illustration of the cultural adaptations for Bangladesh and The Gambia for this paradigm developed in the UK). Responses of 6-month-old (n = 85) and 36-month-old (n = 105) infants living in this low-resource environment replicate findings from the previous studies: social selectivity is present at both time points, and it is more localized in the older infants. Furthermore, correlations between these responses and psychosocial risks, such as maternal education, maternal stress and the caregiving environment, are also reported.

Wijeakumar et al. report findings from a Globalfnirs study investigating early adversity in rural India, focusing on the impact on the functional brain networks underlying visual working memory [41]. Forty-two 4- to 48-month-olds were recruited from the most populous region in India, Uttar Pradesh, where children are exposed to poverty-related developmental challenges from early infancy, and thus represent a population relatively similar to that of the above-mentioned study. The main findings replicate the results of a cohort from mid-west USA (recruited for the study at 4 months (n = 16); 12 months (n = 19); and 24 months (n = 22)) studied with the same protocol; in both cohorts localized activation is reported in the frontal cortex. In line with [38], correlations with psychosocial adversity reveal that children of mothers with lower educational levels show weaker brain activity and poorer distraction suppression. In ongoing work there is also an overlap in shared paradigms between this study and the BEAN and BRIGHT projects.

## 4. Implementing fNIRS in LMICs

Several recent reviews underline challenges specifically related to infants that need to be considered when running studies or analyzing data [42,43]. For instance, positioning the headgear can be tricky, as there is usually a very limited time window of participant tolerance for adjustments. This, on the other hand, serves as incentive for improvement of the headgear design. Further, the brain undergoes a rapid transformation within the first few years of life. This transformation is not only reflected in an increase in size, but also in a structural adjustment of the relative position of the lobes. Therefore, to identify the brain regions where the activation originates, it is necessary to co-register the headgear onto age-appropriate anatomical atlases [44,45]. More sophisticated analyses, such as image reconstruction of the data into 3D models of activation (similarly to functional MRI), require information about the underlying tissue composition, which further highlights the need for population specific and age-appropriate atlases. Work in this direction has begun, with head models now available at the neonatal stage [46]. Models covering later developmental stages across the first two years of life are under development in our group [47]. Finally, although fNIRS does tolerate some movement from participants, when this is excessive, it can interfere with the data acquisition. There are currently a number of options for processing motion artifacts, which have been thoroughly tested with semi-simulated and real adult data [48,49]. However, the parameters used in these analysis pipelines need to be adjusted to the characteristics of infant data. Guidelines on how to fine-tune algorithms and assess their performance are a useful resource [50,51].

fNIRS offers considerable adaptability to a variety of environmental conditions, participant needs and characteristics, and data acquisition protocols. However, as it is starting to be used outside the fully resourced laboratory set-ups in high-resource settings, additional issues need to be taken into consideration for successful data collection. The intricacies of setting up a novel study to investigate language and reading in children of rural Côte d’Ivoire are summarized in a recently published article [52]. Although it is very specific to the study described, its recommendations can be adapted for other low-resource environments. In fact, many of these resonate with methods being implemented within the framework of the BRIGHT project with younger participants [53]. This section summarizes the most relevant ones.

### 4.1 Equipment

Global health studies typically involve large numbers of participants often studied longitudinally, hence imposing intensive schedules for system usage. In the BRIGHT project, testing schedules have required studying up to four infants a day, seven days a week for over two years. To minimize data loss, research assistants and field workers were trained to monitor the system’s performance and detect potential problems with the acquisition, and were also instructed to implement basic repairs.

Extreme heat and humidity can modify the performance of certain materials. In the BRIGHT project, the 3D-printed fNIRS sensor arrays have experienced more failures during testing in The Gambia than in the UK. Training research assistants and field workers to repair the arrays and ensuring a steady supply of array materials has helped avoid interruptions to the testing schedule.

### 4.2 Site Infrastructure

Testing in LMICs may require travelling to remote areas that can be difficult to reach due to an under-developed transport infrastructure. In the case of the BRIGHT project, the equipment was transported to the rural village of Keneba, about 3 h away from the more developed coastal region of The Gambia, partly on an un-paved road. The fNIRS system was packed into flight cases with cutout foam to cushion it for protection during the journey. In the same cases extra fiber bundles and spare parts for emergency repairs were also safely transported.

Air conditioning (a/c) units may be less available in LMIC settings. With no a/c, heat and humidity may increase discomfort on the participants, who can become restless and diminish their willingness to participate in the study. In turn, an increase in sweating can affect the contact between the optodes and the skin, making the skin more slippery and hence increasing the likelihood of a shift in the source/detector array position during data acquisition. This can considerably increase the presence of motion artifacts in the data. Where a/c units are not available, shortened or sequential fNIRS sessions can mitigate these effects.

In addition, in regions with strong weather seasonality, excessive dust during the dry season necessitates systematic cleaning of the testing areas and the use of dust covers to prevent damage to the equipment.

Instability and unreliability of the electrical supply can be an issue in resource-poor settings and this can damage the electronics of fNIRS systems. Installing uninterrupted power supply (UPS) units that protect against power fluctuations and outages can be used to mitigate these issues.

Unreliable internet communication between the sites where data are acquired and the centers where the protocols are developed requires careful planning of protocol updates. On the one hand, software and stimuli files need to be shipped from the development sites; on the other hand, pilot data need to be checked and feedback sent back to the testing teams (in circumstances when the team acquiring the data is unable to conduct quality control checks). Moreover, if datasets are analyzed away from the collection sites, files need to be transferred from collection to analysis sites. All these can be difficult processes as internet bandwidth may struggle with large files. The fNIRS tasks of the BRIGHT project, for example, involve acquiring over 160 files in total across all time points for each participant. This requires an average of 450 MB of data (including NIRS data, infant photos, and videos for behavioral coding) to be transferred per participant at each of the six time points at which fNIRS is performed. A dual protocol has been designed to ensure the integrity of the data transfer. As a standard procedure, a Secured File Transfer Protocol (SFTP) server functions as a bridge between sites. Alternatively, whenever there is unreliable internet coverage, the data can be shipped in batches on portable encrypted hard drives that travel between sites. In addition, the data is stored and backed-up locally on site in a separate location to the acquisition computers.

Running infant fNIRS studies in LMICs requires no greater number of staff per session than it would in Western labs. However we strongly encourage anyone running studies in remote locations to, in addition, create and maintain the post of “site supervisor”; this should be someone with fNIRS experience that can oversee the running of the sessions and liaise with the principal investigators in the project to make adjustments to the protocol to streamline testing. Moreover, this site supervisor could organize and run on-site training sessions for other, less experienced testers. In addition, the site supervisor could perform, and train others in, basic repairs.

### 4.3 Experimental Protocols

Similar to other imaging modalities and behavioral assessments [31], experimental protocols need to be culturally appropriate. This means that any stimuli—including images, videos or spoken language—needtobepresentedbyactorsrepresentativeoftheethnicityandlanguageoftheparticipants, and toys and other objects need to characterize those of their familiar context (see Figure 1 and details of the adaptations for the fNIRS protocols in [53]). It is worth noting that adaptations for neuroimaging are substantially quicker to apply than for behavioral studies, interviews or questionnaires: neuroimaging protocols may only need minor modifications of either visual or auditory stimuli (for an example of a behavioral adaptation see [31]). Nevertheless, all adaptations need to be designed with the objective of eliciting the same type of response in all populations.

### 4.4 Engagement of the Local Community

Part of the success of any study with infants resides in establishing and maintaining communication and engagement with the local community from which the participants are drawn. Transparency in the objective of the project and its methods (technology and experimental protocols) helps build trust and cooperation with participating families. Relaxed and trusting parents and/or family members bringing infants for an fNIRS session are a first step to acquiring good data. To achieve this, families in any context need to be reassured about the safety of the technology, and understand fully what is expected from them and what they can expect to happen during the sessions. Hiring and training local research assistants to explain the study, give instructions, and reassure the families using their own language, fills the potential gap that could open between the project and the community due to language and cultural barriers.

An intensive testing regime requires teams of research assistants trained in running the sessions, maintaining the systems and ensuring the quality of the data collected. At the start of the BRIGHT project, researchers from UCL and Birkbeck travelled to The Gambia to conduct training sessions with the NIRS system, and the first studies were run under supervision. Training was mainly done on site, and, occasionally, via remote connection. The local teams were also trained to monitor data quality in collaboration with researchers based in the UK. This has helped build capacity in the local community, where studies can now be run without the need for continuous supervision.

## 5. Conclusions

In summary, results from recent fNIRS studies in resource-poor regions demonstrate that (i) the technology can be used to study cognitive development in a range of settings; (ii) paradigms can be adapted to provide cross-cultural comparisons; (iii) markers of brain function previously identified in high-income infant populations can also help understand development in LMICs; and (iv) fNIRS has the potential to help disentangle the complex interactions between multiple poverty-driven risk factors on children’s developmental outcomes.

These studies also highlight where future innovations could further propel the use of fNIRS in global health studies. Firstly, although the three studies with data from LMICs mentioned here [37,38,41] present three different approaches to the anatomical identification of the active brain regions, all three methods are based on age-matched MRI templates from the Neurodevelopmental MRI Database [54] (which provides age-specific reference data from 2 weeks to 89 years of age, compiled at the University of South Carolina, USA). One of the challenges faced by longitudinal studies is the accurate calculation of the sensitivity maps for photon distribution in the tissue types probed by the fNIRS sensors for different head sizes. Image reconstruction methods based on models of light propagation models require tissue-identification templates to resolve the forward model (as shown in [41]). The method would benefit from the use of the most appropriate source of anatomical information. Future work should investigate whether population-matched templates in co-registration and image-reconstruction methods would provide more accurate results. In 2015, the first comprehensive template for Chinese children (7 to 16 years of age) was published [55], which represents a first step in this direction. Secondly, the use of shared protocols (as in the case of the BRIGHT and BEAN projects) facilitates direct comparison of brain function markers across populations. Replicating protocols in a variety of populations affected by different risk factors may elucidate which are the most robust biomarkers of adversity and targets for intervention.

Finally, ongoing technological developments in the usability of fNIRS hardware and software align with further applications in global health projects. One of the most promising steps forward has been the development of miniaturized, fibreless wearable systems that enable data collection under very naturalistic circumstances [56]. This is a highly desirable feature for infant and toddler studies and will extend ambitions for how developmental changes in brain function, both typical and atypical, can be assessed in low-resource settings.
